# Population genomics and distribution modeling revealed the history and suggested a possible future of the endemic *Agave aurea* (Asparagaceae) complex in the Baja California Peninsula

**DOI:** 10.1002/ece3.70027

**Published:** 2024-07-24

**Authors:** Anastasia Klimova, Jesús Gutíerrez‐Rivera, Alfredo Ortega‐Rubio, Luis E. Eguiarte

**Affiliations:** ^1^ Centro de Investigaciones Biológicas del Noroeste S.C. La Paz Mexico; ^2^ Departamento de Ecología Evolutiva Instituto de Ecología, Universidad Nacional Autónoma de México Ciudad de México Mexico

**Keywords:** Agavoideae, Baja California Peninsula, climate change, genomic diversity, pollinators, Sonoran Desert

## Abstract

*Agaves* are an outstanding arid‐adapted group of species that provide a unique chance to study the influence of multiple potential factors (i.e., geological and ecological) on plant population structure and diversification in the heterogeneous environment of the Baja California Peninsula. However, relatively little is known about the phylogeography of the endemic agave species of this region. Herein, we used over 10,000 single‐nucleotide polymorphisms (SNPs) and spatial data from the *Agave aurea* species complex (i.e., *A. aurea* ssp. *aurea*, *A. aurea* ssp. *promontorii*, and *A. aurea* var. *capensis*) to resolve genetic relationships within this complex and uncover fine‐scale population structure, diversity patterns, and their potential underlying drivers. Analyses resolved low genetic structure within this complex, suggesting that *A. aurea* is more likely to represent several closely related populations than separate species or varieties/subspecies. We found that geographical and historical ecological characteristics—including precipitation, latitude, and past climatic fluctuations—have played an important role in the spatial distribution of diversity and structure in *A. aurea*. Finally, species distribution modeling results suggested that climate change will become critical in the extinction risk of *A. aurea*, with the northernmost population being particularly vulnerable. The low population genetic structure found in *A. aurea* is consistent with agave's life history, and it is probably related to continuity of distribution, relatively low habitat fragmentation, and dispersion by pollinators. Together, these findings have important implications for management and conservation programs in agave, such as creating and evaluating protected areas and translocating and augmentation of particular populations.

## Graphical abstract

Little is known about the phylogeography of the endemic agave species of the Baja California Peninsula. Using over 10,000 single‐nucleotide polymorphisms (SNPs) and spatial data from the *Agave aurea* species complex (i.e., *A. aurea* ssp. *aurea*, *A. aurea* ssp. *promontorii*, and *A. aurea* var. *capensis*), we determined genetic relationships within this complex and uncovered fine‐scale population structure, diversity patterns, and their potential underlying drivers. Analyses resolved low genetic structure within this complex, consistent with agave's life history, suggesting that *A. aurea* is more likely to represent several closely related populations than separate species or varieties/subspecies. Geography and historical ecological events have played an important role in the spatial distribution of diversity and structure in *A. aurea*.

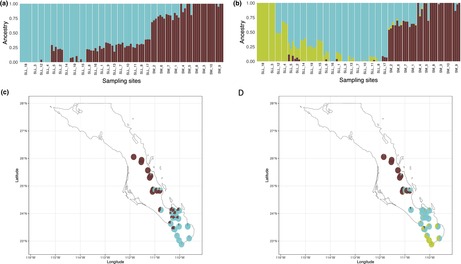

## 1 INTRODUCTION

Arid lands are one of the most widespread ecosystems worldwide (Prăvălie, 2016; Ward, 2016), and due to anthropogenic activities, their borders are expanding (Liu & Xue, 2020; Mirzabaev et al., 2019). Despite the harsh environment, deserts are known for a large number of endemic species and high plant functional diversity (Maestre et al., 2012, 2021; Scherson et al., 2020). Moreover, drylands are crucial in global biogeochemical cycles and Earth's energy balance (Jickells et al., 2005; Okin et al., 2004).

Although living in deserts is highly stressful, some plant groups have evolved unique morphological, physiological, and behavioral adaptations, including crassulacean acid metabolism (CAM) photosynthesis, delayed germination, clonality, extended shallow root system, succulence, and production of particular heat‐shock proteins, which allow them to thrive in the harsh conditions of arid and semiarid areas (Smith, 1997; Wickens, 1998; Ward, 2016). Nevertheless, due to the long generation time of many species, slow plant turnover, slow regeneration, and significant reliance on plant–plant interactions (“nurse plants”), desert flora may be particularly sensitive to the projected increase in temperature and aridity (Brown et al., 2023; Cody, 2000; Ravi et al., 2021). Moreover, it is quite possible that desert plant species already operate close to their physiological limits (Hantson et al., 2021; Madsen‐Hepp et al., 2023). For instance, a recent simulation study suggested that climate change will be a primary cause of cactus extinction risk, with over 60% of species assessed being negatively impacted (Pillet et al., 2022). Climate change is also shifting the balance in plant–soil interactions when an increase in aridity reduces plant fungal symbionts and substantially increases the proportion of fungal pathogens, which negatively impacts plant's fitness (Maestre et al., 2021; Pugnaire et al., 2019).

Some of the iconic species of the deserts of North America are members of the Agavoideae (Asparagaceae), including agaves and yuccas (Gentry, 1978, 1982). *Agave* is a genus of monocotyledons native to the arid lands of North America (Eguiarte et al., 2021). Agaves flourish in arid and semiarid areas, and in many regions, they are dominant species that provide food and shelter for many organisms (Gentry, 1978, 1982). The genus's great ecological success has been linked to crassulacean acid metabolism (CAM) and the generalist pollination system (Eguiarte et al., 2021). Birds, bees, and flies pollinate many agave species during the day, and nectar‐feeding bats pollinate them at night (Rocha et al., 2006). In Mesoamerica and Aridoamerica, the genus also has enormous cultural and economic importance (Alducin‐Martínez et al., 2022; Gentry, 1982). Nevertheless, slow growth, low reproductive rates, the importance of plant–plant interactions (i.e., “nurse plants effect”), and plant–pollinator interactions make agaves especially susceptible to environmental disturbances and possibly to climate change (Gómez‐Ruiz & Lacher, 2019; Martínez‐Palacios et al., 1999).

Recently (~7 Mya and ~2.5 Mya), agaves experienced two bursts of evolutionary diversification that resulted in many endemic and microendemic species, with countless forms of leaves, rosettes, and inflorescences (Eguiarte et al., 2021; Gentry, 1978, 1982; Good‐Avila et al., 2006; Jiménez‐Barrón et al., 2020). On the Baja California Peninsula (BCP hereafter), for example, a total of 23 *Agave* taxa are found, with 22 of them being endemic (Trelease, 1911; Webb & Starr, 2015). Surprisingly, the rich diversity of agaves in the BCP has been little studied (Gentry, 1978; Webb & Starr, 2015). For example, almost all *Agave* taxa in BCP represent species/subspecies complexes with unclear geographical boundaries or genetic relationships between and within them (Navarro‐Quezada et al., 2003; Webb & Starr, 2015).

The high biological diversity and endemism levels of flora on the BCP are thought to arise from the prolonged isolation, peculiar geography of the peninsula, and its high landscape heterogeneity (Garcillán et al., 2010; Grismer, 2000; Riddle et al., 2000; Riemann & Ezcurra, 2007; Van Devender, 1990). The BCP lies between the Pacific Ocean and the Gulf of California; it is one of the longest and the most isolated peninsulas, reaching a length of approximately 1300 km (Dolby et al., 2015). The BCP is mostly arid (~75%) and forms part of the Sonoran Desert, with readily recognized vegetation including different species of agave, columnar cacti (e.g., *Pachycereus pringlei* and *Stenocereus thurberi*), yuccas, and the Boojum tree (*Fouquieria columnaris*) (Riemann & Ezcurra, 2007). BCP desert is relatively young and thought to have originated during the late Miocene and the Pliocene, 5–10 million years ago, with the modern warm‐desert vegetation becoming extensive only approximately 6000–12,000 years ago, after the end of the last glacial period (Axelrod, 1978; Frenzel, 2005; Raven & Axelrod, 1978). As a consequence, a signal of relatively recent northward and southward expansion from refugia has been observed along the BCP in arid‐adapted and succulent plant taxa (De la Rosa‐Conroy et al., 2019; Garrick et al., 2009; Gutiérrez‐Flores et al., 2016; Nason et al., 2002).

**FIGURE 1:**
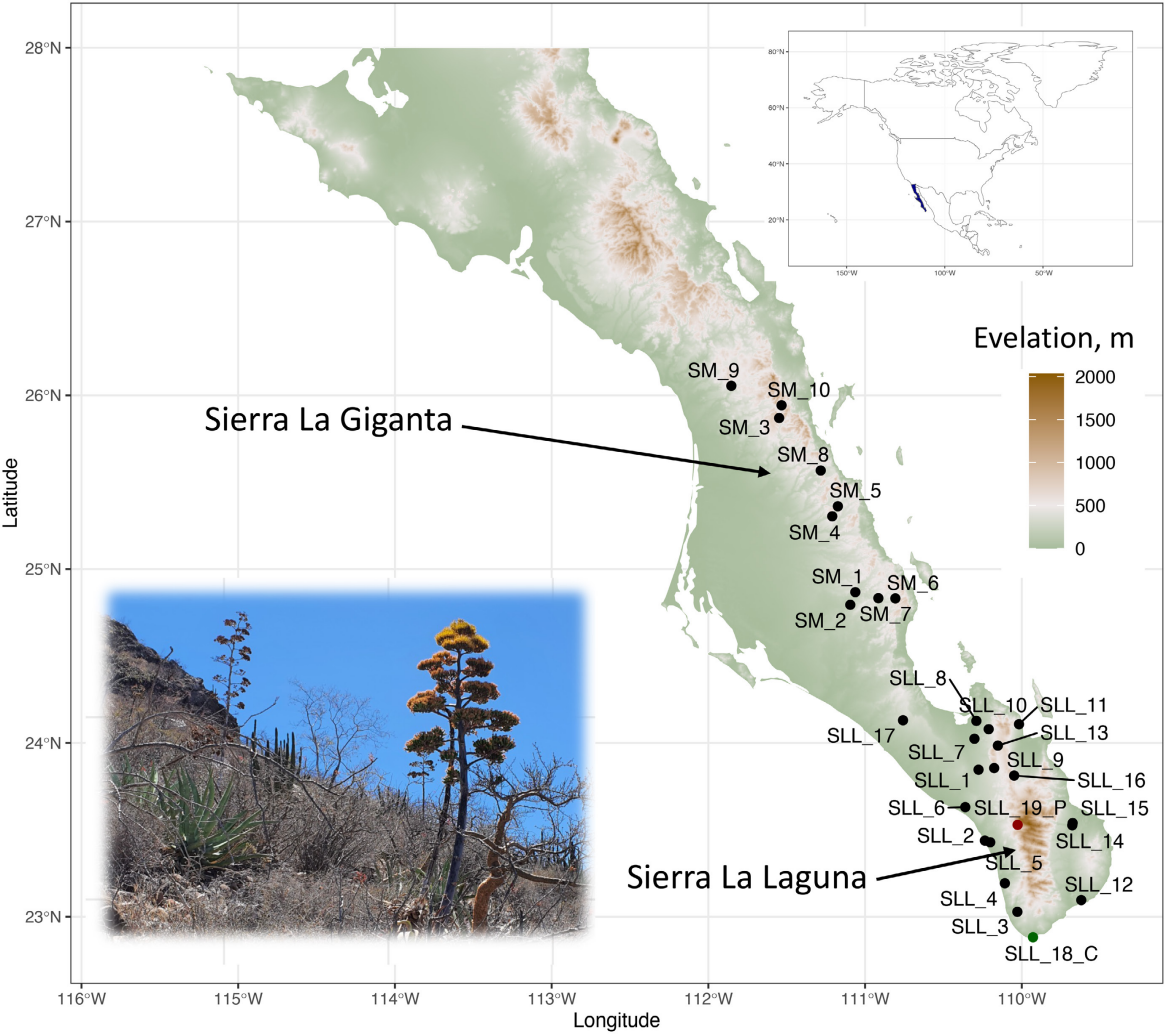
Map of the southern Baja California Peninsula, Mexico, with a background representing the topography of the area and black dots representing 29 sample site locations of *Agave aurea* sensu Webb and Starr (2015). Sampling site abbreviations can be found in Table S1; the subspecies of *A. aurea* var. *capensis* and *A. aurea* ssp. *promontorii* are coded and colored as SLL_18_C (dark green) and SLL_19_P (dark red), respectively. Inset at the left corner is a picture of *A. aurea* spp. *aurea* collected in Sierra La Giganta. Inset at the right corner is a map of North America, with the Baja California Peninsula highlighted in dark blue.

Among the unique floristic diversity of BCP, *Agave aurea* stands out (Figure 1). In the BCP, *A. aurea* is the only representative of the Campaniflorae section (Gentry, 1978, 1982; Webb & Starr, 2015). The species is found on the west side of the Sierra La Giganta, extending south to the Sierra La Laguna and Cabo San Lucas (Webb & Starr, 2015). It is a relatively large plant with a tall, showy inflorescence that can reach 8 meters. The plant used to be harvested by indigenous people as a source of food and fiber. Nowadays, it is sometimes used to make a distilled alcoholic beverage (a type of mezcal) and as an ornamental plant. The plant is said to have been trialed as a commercial source of fiber, but yields were too low (Gentry, 1978).

Currently, *A. aurea* is considered to be a species complex, with the taxonomic status of varieties/subspecies being under debate (Webb & Starr, 2015). Initially, based on flower characteristics, three separate species, *Agave aurea*, *Agave capensis*, and *Agave promontorii*, were described (see Gentry, 1978, 1982). Recently, Webb and Starr (2015) indicated that, due to the similarity in vegetative characteristics and the differences mainly related to size and the propensity for clonal reproduction, these three species should be reduced to the subspecies or varieties level. No genetic studies have been done to clarify taxonomic uncertainties within the *A. aurea* complex, yet.

Of the three varieties or subspecies sensu Webb and Starr (2015), *A. aurea* ssp. *aurea* is the most common and abundant, extending from the western slopes of the Sierra La Giganta to the Sierra La Laguna in the Cape Region. It is easily recognized by the long, narrow green leaves that arch to form an open rosette and by the bright yellow flowers (Figure 1). *Agave aurea* var. *capensis* is a smaller plant with narrower leaves and differs from other varieties mainly because it proliferates by axillary sprouting, creating large groups of plants. *Agave aurea* var. *capensis* has restricted geographic distribution and can only be found on the peninsula's southern tip. *Agave aurea* ssp. *promontorii*, on the other hand, is a large plant restricted to the northern Sierra La Laguna at elevations of 900–1800 m. The genetic relationships between these varieties and their exact geographic boundaries are unclear and need further investigation (see Webb & Starr, 2015).

In addition to the uncertainty in species delimitation, there is a growing concern for the conservation of the flora and fauna of the BCP (Benavides et al., 2020; Dávila et al., 2022; Klimova, Gutiérrez‐Rivera, et al., 2022; Klimova, Mondragón, et al., 2022; Riemann & Ezcurra, 2005; Vanderplank et al., 2014). Like other arid areas around the globe, Baja California has been heavily influenced by anthropogenic disturbances, such as overgrazing by free‐roaming livestock, off‐road recreational vehicles, agriculture, and climate change (Riemann & Ezcurra, 2005; Wehncke et al., 2014). In recent decades, rapid development and human population growth have greatly intensified threats to BCP's ecosystems, especially to endemic and endangered species (IUCN, 2023; SEMARNAT, 2010; Wehncke et al., 2014). Among human‐induced biodiversity threats, climate change is predicted to play an increasingly important role in biodiversity decline (Gao et al., 2020; Pinsky & Fredston, 2022; Urban, 2015). Moreover, climate change has already had a significant negative impact on Sonoran Desert vegetation, particularly on its xeric portion, leading to a substantial decrease in vegetation cover (Hantson et al., 2021).

To address the unresolved issues described above, we combined genome‐wide SNPs and species distribution modeling (SDM) with a thorough sampling of the *Agave aurea* complex in the BCP. The data were analyzed on two levels. First, we tried to resolve genetic relationships within this complex and, from there, delimit the geographic boundaries of each group. We then focused on fine‐scale genetic analyses, uncovering the population structure and diversity patterns of *A. aurea* and investigating their potential underlying drivers. Second, we tried to determine how the future potential distribution of the *A. aurea* complex will be altered under different climate change scenarios. Our main working hypotheses were: (1) We expected to find genomic support for at least some currently recognized subspecies/varieties within *A. aurea* complex. (2) As bats are an important mediator of pollen dispersal in agaves, and agaves, in general, present low genetic differentiation within species, we hypothesized that for *A. aurea*, pollen dispersal would not be restricted within geographic regions, which should be reflected in overall shallow population structure. (3) We expected that the distribution of genetic diversity and differentiation of *A. aurea* would be related to the geography, ecological, and climatic history of BCP. (4) Due to the projected aridification of BCP, we hypothesized that the future potential distribution of *A. aurea* will be negatively affected by climate change.

## 2 MATERIALS AND METHODS

### 2.1 Sample collection

Fresh leave tissue samples were collected from 29 geographic locations across the southern part of the BCP that represent the complete distribution range of *Agave aurea* sensu Webb and Starr (2015) (Figure 1, Table S1). All three recognized subspecies (Gentry, 1978; Webb & Starr, 2015) were included in the analysis. For *Agave aurea* var. *capensis*, besides a type locality, several more sites extracted from the Global Biodiversity Information Facility (GBIF, 2023) were visited, but only plants morphologically similar to *Agave aurea* ssp. *aurea* were found (relatively large plants that did not form clonal clusters). *Plants were assigned to subspecies based on their morphological characteristics and the geographic locality where they were collected* (Gentry, 1978; Webb & Starr, 2015).

Upon collection, samples were preserved in paper bags and kept away from heat and sunlight; once at the laboratory, samples were kept at −20°C until DNA extraction. All the specimens were collected during fieldwork performed in 2023.

### 2.2 DNA extraction and RADSeq

Genomic DNA from 98 individuals of *Agave aurea* sensu Webb and Starr (2015) was extracted using a modified hexadecyltrimethylammonium bromide (CTAB) protocol from the frozen leaf tissue disrupted with liquid nitrogen (Doyle & Doyle, 1987; Klimova, Gutiérrez‐Rivera, et al., 2022; Klimova, Mondragón, et al., 2022). The DNA quality was checked on a 1% agarose gel. Samples of adequate quality and quantity were sent to the University of Wisconsin Gene Core for RAD‐Seq library preparation (Andrews et al., 2016; Elshire et al., 2011) using two methylation‐sensitive restriction enzymes (PstI/MspI) and sequencing on the Illumina NovaSeq 2 × 150 platform (Illumina, San Diego, CA, USA).

The resulting paired‐end reads were assessed for quality using FastQC (Andrews, 2010) and filtered using the fastp (Chen et al., 2018). We removed adapters, sequences shorter than 55 bp, reads with over five N, low‐quality sequences, and trimmed poly G and poly X tails. We also filtered low‐complexity reads, where the complexity was defined as the percentage of a base that is different from its next base (base[*i*]! = base[*i* + 1]) (Chen et al., 2018). Filtered reads were demultiplexed using the process_radtags function in STACKS v1.41 (Catchen et al., 2013; Rochette et al., 2019) and mapped to the *Agave tequilana* transcriptome (GAHU00000000.1; Gross et al., 2013) using Burrows‐Wheeler Aligner (BWA) v0.7.13 (Li & Durbin, 2009). The resulting Sequence Alignment Map (SAM) files were converted to Binary Alignment Map (BAM) format, sorted by coordinates, and indexed using SAMtools (Danecek et al., 2021). SNPs were called using bcftools (Danecek et al., 2021).

The raw genotypes were filtered using VCFtools v0.1.16 (Danecek et al., 2011). We removed SNPs with a minor allele count of <8, a genotyping rate of less than 90%, a maximum mean depth of 150, a minimum mean depth of 10, and a maximum number of alleles of 2. We removed loci that deviated from Hardy–Weinberg equilibrium (*p* < .05, after Bonferroni correction). Finally, we removed loci in linkage disequilibrium (*r* ^2^ > 0.2) using PLINK v1.90b6.21 (Chang et al., 2015).

### 2.3 Population structure

To investigate population structure and to understand the relationships within the *A. aurea* complex sensu Webb and Starr (2015), we used several complementary approaches. We conducted a principal component analysis (PCA) using the R package SNPRelate (Zheng et al., 2012). We estimated individual admixture proportions using ADMIXTURE (Alexander et al., 2009; Alexander & Lange, 2011). Admixture runs were performed for ancestry clusters (*K*) ranging from 1 to 10, with 10 runs for each *K* value. The optimal number of clusters was identified based on the lowest cross‐validation error and visualized using R. We also used fineRADstructure, a Bayesian clustering approach that utilizes haplotype linkage information and searches the most recent coalescence (common ancestry) among the sampled individuals (Malinsky et al., 2018). A “coancestry matrix” of *A. aurea* specimens was generated using STACKS's ‘population’ module (Catchen et al., 2013). We subsequently used 10,000,000 Markov chain Monte Carlo (MCMC) iterations with a burn‐in of 5,000,000 and sampling occurring every 10,000 iterations. A tree was constructed with 100,000 hill‐climbing iterations, and the results were visualized using the script FINERADSTRUCTUREPLOT.R, which is available at https://github.com/millanek/fineRADstructure. We also reconstructed the genealogical relationships among *A. aurea* individuals using an neighbor‐joining (NJ) tree estimated using the bitwise.dist function within the R package poppr and aboot function, using 1000 bootstrap replicates (Kamvar et al., 2015). The unrooted NJ tree among sampling sites based on the Nei genetic distance was estimated with the R packages StAMPP (Statistical Analysis of Mixed‐Ploidy Populations) and ape (Paradis & Schliep, 2018; Pembleton et al., 2013). Finally, we estimated pairwise genetic differentiation among subspecies, sampling sites, and geographic regions, using Weir and Cockerham *F*_ST_ values calculated (Weir & Cockerham, 1984) in the R package StAMPP. Confidence intervals and *p‐*values were estimated based on bootstrap resampling of individuals 100 times.

### 2.4 Landscape genetics

Geographic isolation and dispersal barriers are known to contribute to the geographic structuring of genetic variation in many organisms (Bradburd et al., 2013; Loveless & Hamrick, 1984; Wright, 1949). Therefore, we examined the relationship between genetic and geographic distance between all pairs of sampling sites. Genetic distance was based on pairwise *F*_ST_ obtained using the R package StAMPP. Geographic great‐circle distance among sampling sites was calculated using the Geographic Distance Matrix Generator version 1.2.3 (Ersts, 2013). The significance of genetic and geographic distance association was calculated using Mantel tests with the R package ade4 (Dray & Dufour, 2007) according to the method proposed by Rousset (1997), which is based on the FST/(1 − FST) and the natural logarithm of geographic distance (ln).

The divergence can also be explained by local adaptation that will show a correlation between genetic and environmental distances (Frankham et al., 2002). We used Mantel and partial Mantel tests as implemented in the R package VEGAN 2.4‐0 (Oksanen et al., 2014) to test for correlations between genetic and environmental distances, the latter being generated using the “dist” function in R from all the 19 bioclimatic variables downloaded from WorldClim v.2 (Hijmans et al., 2005) as a set of raster layers. Mantel tests were performed between each genetic and environmental distance matrix, and these analyses were also repeated as partial Mantel tests controlling for geographic distance. Statistical significance was determined using Pearson's tests based on 10,000 permutations. To avoid collinearity among ecological variables, we performed a Mantel test between the climatic variables significantly correlated with genetic differentiation and used only those variables that did not correlate with each other. Between highly correlated variables, we chose one with the highest *r*‐statistic and the lowest *p*‐value (Table S3).

Finally, we used the clustering method implemented in TESS3R that considers genetic and geographic data to determine the most probable number of clusters (*K*) in a geographic space (Caye et al., 2015). We tested *K* = 1 to 10 with 30 replicates of each *K* and kept the most supported model (i.e., “best,” based on cross‐entropy scores) within each of the 30 replicates. Locations on the map were colored according to the resulting dominant ancestry cluster.

### 2.5 Genome‐wide diversity

To assess levels of genetic diversity within *A. aurea* sensu Webb and Starr (2015), we estimated multilocus heterozygosity (MLH) using the R package inbreedR (Stoffel et al., 2016) and the inbreeding indices *F*_IS_ and *Fhat3* using PLINK 2.0 (Chang et al., 2015). These diversity metrics were calculated at the individual level. To better understand the spatial distribution of genetic diversity, we plotted the diversity estimates on a map using the R package ggplot2 (Wickham, 2009). We then explored whether geographical variables (i.e., elevation and latitude as predictive variables) were related to the levels of genetic diversity by using simple and quadratic linear models (LMs) in the R package stats (R Core Team, 2021). We also estimated the number of private alleles for each subspecies/geographic region using the R package poppr.

### 2.6 Species distribution modeling (SDM)

Occurrence data for *A. aurea* sensu Webb and Starr (2015) were downloaded from the Global Biodiversity Information Facility (GBIF, 2021) and supplemented with our field surveys. We excluded locations that fell into the ocean or in human settlements, old data (older than 1970), and coordinates with an uncertainty of over 200 m; data were also filtered within the BIOMOD2 package (Thuiller, 2003; Thuiller et al., 2009) using the filter. raster function. In total, 128 occurrence points were retained.

We used the current climatic variables at a spatial resolution of 30 arc‐s from the WorldClim to estimate the current potential distribution of *A. aurea* sensu Webb and Starr (2015). To avoid collinearity among bioclimatic variables, we used Pearson's correlation analysis to choose only one variable from each pair of strongly associated variables (i.e., *r* > 0.75 or −0.75). A total of 10 variables were retained after correlation analysis (Table S2).

For predicting the future potential distribution of *A. aurea*, we used the Coupled Model Intercomparison Project (CMIP6) to access the climate models based on two representative shared socioeconomic pathways (SSP 245 and SSP 585) for a time period from 2061 to 2080. Future climate models rely on diverse sets of codes and are parameterized with slightly different conditions; therefore, as suggested by Knutti et al. (2013) and Sanderson et al. (2015), we selected three dissimilar models (Australian Community Climate and Earth System Simulator‐Earth System Model 1.5 (ACCESS‐ESM1‐5), Model for Interdisciplinary Research on Climate, sixth version (MIROC6), and Max‐Planck Institute‐Earth System Model version 1.2 low resolution (MPI‐ESM1‐2‐LR)). All climatic data were downloaded using R package *geodata* (R Core Team, 2021). The same set of environmental variables used to estimate the current distribution of *A. aurea* sensu Webb and Starr (2015) was also used to predict its future potential distribution.

We performed the ensemble distribution modeling using Generalized Boosted Models (GBM) (Ridgeway, 1999) and Random Forest (RF) (Breiman, 2001) algorithms. The distribution modeling requires the presence and absence of data; we, therefore, randomly generated 1000 pseudo‐absence points and five pseudo‐absence data sets (Guisan et al., 2017). We built the models using 80% of the data (training set) and evaluated the model performance with the rest of the 20% of the data (evaluation set). We ran each of the models 10 times. We used two evaluation metrics to determine the accuracy of the models: the area under the curve (AUC) of receiver operating characteristics (ROC) and true skills statistics (TSS) (Khan & Verma, 2022; Rather et al., 2022). To visualize and measure the range change for *A. aurea* sensu Webb and Starr (2015) under future climatic conditions, we used the range–size function implemented in the BIOMOD2 package.

We also used SDM to predict climatically suitable areas for *A. aurea* sensu Webb and Starr (2015) under two different past time periods (Mid‐Holocene (MH) and Last Glacial Maximum (LGM)). The raster layers were downloaded from WorldClim (Fick & Hijmans, 2017) and two different climatic models were chosen (Community Climate System Model, version 4 (CCSM4) and Max Planck Institute for Meteorology (MPI‐M)‐Earth System Model‐P Model (MPI‐ESM‐P)). We got a resolution of 30 arc‐seconds for Mid‐Holocene data, whereas for LGM, it was 2.5 min. The SDM for past conditions was implemented, as described above.

## 3 RESULTS

We generated RADSeq data for 98 *A. aurea* sensu Webb and Starr (2015) individuals collected from 29 locations that represent the complete distribution range of the species. We included the three subspecies/varieties recognized by Webb and Starr (2015) (Figure 1, Table S1). For *A. aurea* ssp. *aurea*, we included 87 samples, comprising 27 sampling sites from the Cape Region to the Sierra La Giganta. For *A. aurea* ssp. *promontorii* (*n* = 5), we included one site at Sierra La Laguna, and for *A. aurea* var. *capensis* (*n* = 6), one sampling site at the type locality Cerro de la Zeta at the Cape Region of the BCP.

From a total of 547,875 raw SNPs called using the Samtools, after filtering with VCFtools, 10,765 high‐quality SNPs across all subspecies were retained. The mean individual depth among 98 individuals was 57.5 (SD 11.4). The average missingness on a per‐individual basis was 1.4%.

### 3.1 Population structure

**FIGURE 2:**
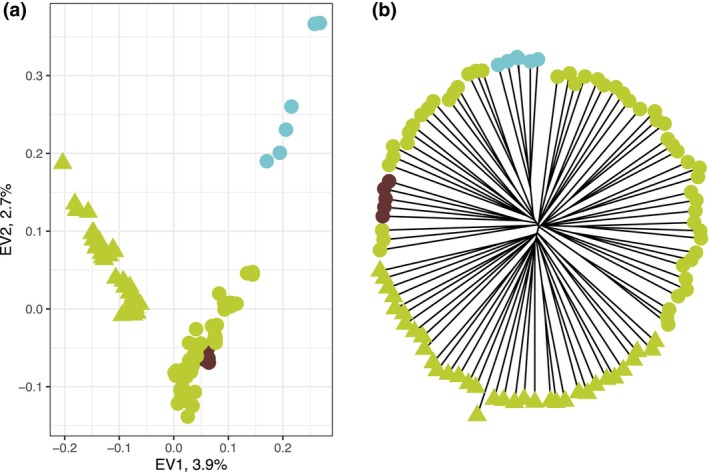
Population genetic structure of *Agave aurea* sensu Webb and Starr (2015) from Baja California Peninsula, Mexico, based on 10,765 genome‐wide SNPs. (a) Principal component analysis (PCA) of the individuals of *A. aurea*. (b) Neighbor‐joining (NJ) network of 98 individuals of *A. aurea*. PCA and NJ tree tips are colored according to the three subspecies of *A. aurea* sensu Webb and Starr (2015), green – *A. aurea* ssp. *aurea*, brown – *A. aurea* ssp. *promontorii*, and blue – *A. aurea* var. *capensis*. Shapes on PCA and NJ tree tips correspond to the mountain ranges from where samples were collected, such as the circle Sierra La Laguna and the triangle Sierra La Giganta.

**FIGURE 3:**
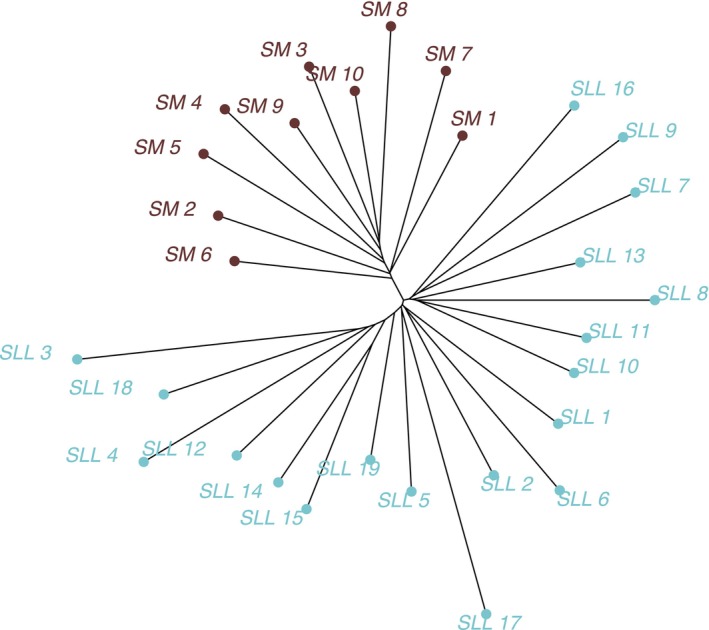
Population genetic structure of *A. aurea* from Baja California Peninsula, Mexico, based on 10,765 genome‐wide SNPs, represented by a Neighbor‐joining (NJ) network of 29 sampling sites. NJ tree tips are colored according to the mountain range: Blue – Sierra La Laguna and brown – Sierra La Giganta. Sampling site abbreviations can be found in Table S1.

**FIGURE 4:**
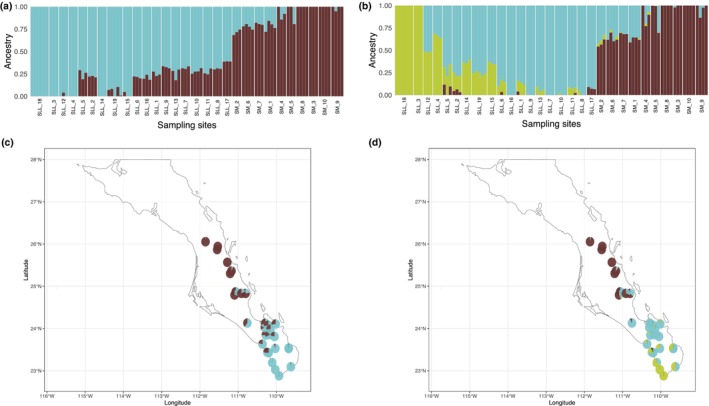
Population genetic structure of the 98 *A. aurea* samples collected in the Baja California Peninsula, Mexico, based on 10,765 SNPs. Bar plots of the individual assignment probabilities (vertical axis) for the number of genetic clusters from *K* = 2 (a) to *K* = 3 (b) inferred using the program ADMIXTURE. Samples were clustered according to sampling sites and arranged from the southernmost sampling site (left) to the northernmost sites (right). Above each Bar plot, the ADMIXTURE *Q*‐values represented as pie charts for each sampling site, for the clustering of *K* = 2 (c) and *K* = 3 (d), plotted on a study area map. Population codes as given in Table S1.

**FIGURE 5:**
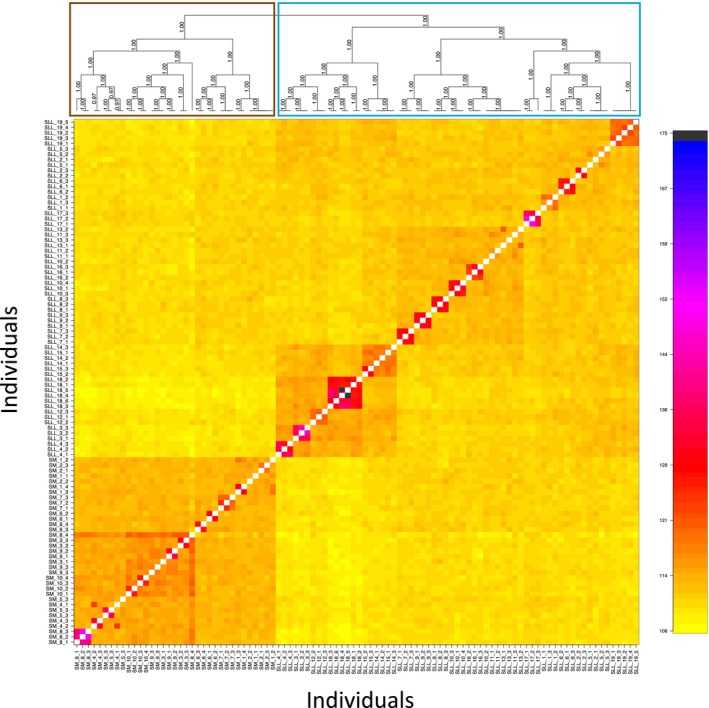
FineRADstructure analysis of haplotype similarity among *A. aurea* specimens. A co‐ancestry matrix was reconstructed using 10,765 SNPs. Colors indicate the scale of relatedness between individuals, with yellow representing low relatedness and blue/black indicating high relatedness. Colored boxes over the phylogram correspond to the two main geographic regions (Sierra La Laguna in blue and Sierra La Giganta in brown). Samples are coded as given in Table S1.

To investigate population differentiation within *A. aurea*, we used several complementary approaches: principal component analysis, NJ tree, ADMIXTURE, pairwise *F*_ST_, and Bayesian clustering in fineradstructure. The overall divergence between varieties was low: *A. aurea* ssp. *aurea* vs. *A. aurea* var. *capensis*, *F*_ST_ = 0.09; *A. aurea* ssp. *aurea* vs. *A. aurea* ssp. *promontorii*, *F*_ST_ = 0.03; and *A. aurea* ssp. *promontorii* vs. *A. aurea* var. *capensis*, *F*_ST_ = 0.14; each estimate was significant (*p* < .001). Although with different sensitivities, all methods agreed that the morphologically described varieties present very shallow genetic divergence among them (Figures 2, 3, 4, 5). For example, it was hard to identify any particular separated genetic group within the individual‐based NJ tree (Figure 2b). There were indications that samples of *A. aurea* var. *capensis* were slightly differentiated from the rest of the specimens, which was also confirmed by PCA. However, the first two principal components explained only 6.65% of the variance (Figure 2a).

The PCA further uncovered previously unrecognized regional structuring related to the geology and geography of the BCP, samples clustered according to the mountain range: southern samples collected on and around Sierra La Laguna vs. northern samples collected on Sierra La Giganta (Figure 2). Nevertheless, the genetic differentiation between mountain ranges was relatively low (*F*_ST_ = 0.03, *p* < .01). These findings were also visible on an individual‐based NJ tree (Figure 2) and were more pronounced when the tree was reconstructed using the populations (sampling sites instead of each specimen, Figure 3).

ADMIXTURE's cross‐validation error (CVE) indicated that the best *K* value for *A. aurea* samples was 2 (Figure S1). When the individual ancestries were plotted (Figure 4), at the best *K* value (*K* = 2), we found a north–south clinal clustering of the samples, with clear geographic structuring into northern (Sierra La Giganta) and southern (Sierra La Laguna and Cape Region) groups. Further partitioning of the samples, *K* = 3, indicated that the three southernmost sampling sites (including the *A. aurea* var. *capensis*, SLL_18) had different genomic ancestry. Moreover, several northernmost sites also had different ancestry, with samples in the middle of the sampled region having mixed ancestry (Figure 4). ADMIXTURE results were confirmed by the among sampling sites *F*_ST_ estimation (Figure S2); the values ranged from *F*_ST_ = 0.03 (between SLL_13 and SLL_11, separated by ~20 km) to *F*_ST_ = 0.26 (SLL_17 and SLL_3, separated by ~141 km).

These patterns were reinforced with the fineradstructure analysis, which pointed to the presence of three main genetic clusters (Figure 5). The first group comprised samples collected in Sierra La Giganta; the second group comprised samples of *A. aurea* var. *capensis* and samples of *A. aurea* ssp. *aurea* (SLL_3, SLL_4, SLL_12, SLL_15, and SLL_14) collected in the southernmost part of Sierra La Laguna; finally, the third cluster comprised samples of *A. aurea* ssp. *promontorii* and *A. aurea* ssp. *aurea* collected roughly on the central and northern parts of the Sierra La Laguna (Figure 5). The co‐ancestry matrix also showed high within‐site relatedness (Figure 5).

### 3.2 Spatial structure

The cross‐validation criterion recovered for *A. aurea* samples using *TESS3R* did not exhibit a minimum value or a plateau (Figure S3), probably reflecting low differentiation within agave populations. Therefore, we plotted the results of different *K* values (from *K* 2 to 3; Figure S4). At *K* = 2, samples were partitioned into groups located approximately south and north of latitude 23°N, with samples of *A. aurea* var. *capensis* (SLL_18) and one site from *A. aurea* ssp. *aurea* (SLL_3) being the only samples with an ancestry of over 99%; the rest of the samples had mixed ancestry (Figure S4a,c). At *K* = 3, we found support for the differentiation between southern and northern (corresponding to the geological break between Sierra La Giganta and Sierra La Laguna mountain ranges) samples, similar to the results observed with PCA and ADMIXTURE (Figure S4b,d). Nevertheless, all the samples presented some degree of mixed ancestry (Figure S4). The third cluster comprised only the specimens belonging to *A. aurea* var. *capensis* (SLL_18) (Figure S4b,d).

Surprisingly, we found a low relationship between genetic *F*_ST_ and geographic distance (Mantel's *r* = 0.17, *p* = .04). Partial Mantel tests revealed significant associations between genetic distance and several environmental variables related to temperature (BIO 2, BIO 4, BIO 6, and BIO 7). Nevertheless, all these variables also presented a high correlation with each other. We decided to keep only the variable with the highest *r*‐statistic and lower *p*‐value (Table S3). Specifically, BIO 4 (Temperature Seasonality (standard deviation × 100)) correlated significantly with genetic distance in *A. aurea* sensu Webb and Starr (2015), even after controlling by geographic distance among samples (Partial Mantel test, *r* = 0.33, *p* = .002).

Taking together all the above population genetic structure and spatial analyses, we can conclude that within *A. aurea* sensu Webb and Starr (2015), there are three main genetic clusters, albeit with low divergence among them: (i) samples of *A. aurea* var. *capensis*, along with several samples of *A. aurea* ssp. *aurea*, restricted to the southernmost region of the Baja California Peninsula; (ii) samples of *A. aurea* ssp. *aurea*, with an affinity to Sierra La Laguna, and samples of *A. aurea* ssp. *promontorii*; and (iii) samples of *A. aurea* ssp. *aurea* distributed at the Sierra La Giganta.

### 3.3 Diversity landscape across subspecies and populations

**FIGURE 6:**
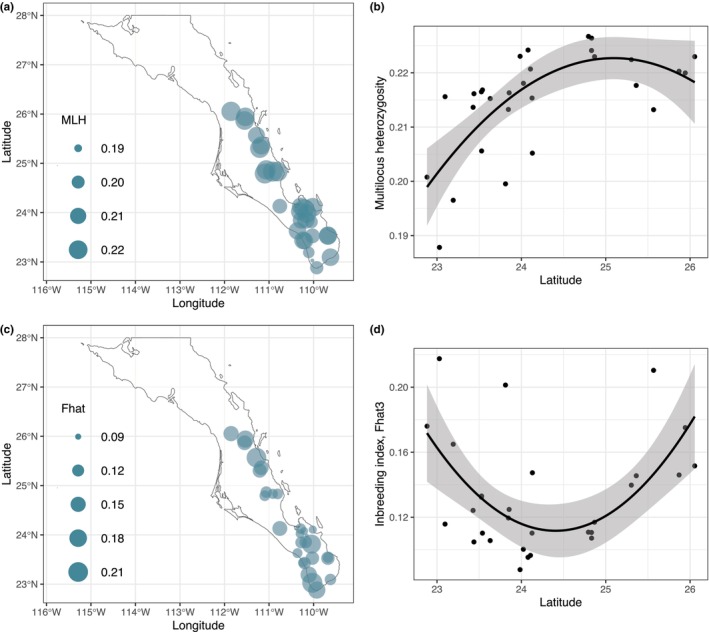
Spatial distribution of individual‐based diversity of *A. aurea* samples. (a) Multilocus heterozygosity and (c) Fhat3 inbreeding index. (b) Relationship (quadratic regression) between MLH and latitude (*R* ^2^ = .46, *p* < .0001) and (d) between Fhat3 and latitude (*R* ^2^ = .3, *p* < .003).

To explore how population structure, geography, and ecological history of the BCP have influenced the genome‐wide variation in *A. aurea* sensu Webb and Starr (2015), we compared individual multilocus heterozygosity and inbreeding indices between subspecies, populations (mountain ranges), and sampling sites. The overall MLH was 0.21 (SD 0.01), with the lowest values (MLH = 0.17) found in individuals from the sampling sites SM_8 and SLL_16 and the highest value (MLH = 0.24) in SLL_13 (Figure 6, Tables S4 and S5). There was a significant (*p* < .005) difference in MLH between mountain ranges, with lower heterozygosity found in Sierra La Laguna, i.e., the southern samples (Figure 6, Table S5). Although Fhat3 and *F*_*IS*_ inbreeding indices were calculated differently, we found similar results for both inbreeding estimates, with low to moderate inbreeding in almost all individuals of *A. aurea*. The mean species‐wide inbreeding index (Fhat3) was 0.14 (SD 0.05), with the lowest values found in samples from SLL_13 (0.05) and the highest inbreeding value found in an individual from SM_8 (0.39). No significant difference was found for the Fhat3 inbreeding index between mountain ranges (Table S6). We also found considerable levels of inbreeding measures by *F*_*IS*_; the mean *F*_*IS*_ was 0.14, ranging from *F*_*IS*_ = 0.05 in a sample from the SLL_13 to *F*_*IS*_ = 0.31 in the SM_8. The inbreeding *F*_*IS*_ was significantly different between mountain ranges, being higher in the Sierra La Laguna, i.e., the southern samples (*p* < .001) (Table S6).

We further explored the relationships between the genetic diversity of *A. aurea* sensu Webb and Starr (2015) populations and BCP's geography and ecological history (Figure 6). The genomic diversity changed significantly with latitude, both in their levels (i.e., MLH vs. latitude *R* ^2^ = .46, *p* < .0001) and in inbreeding (Fhat vs. latitude *R* ^2^ = .3, *p* = .003; *F*_*IS*_ vs. latitude *R* ^2^ = .46, *p* < .001). The correlations were not linear, as lower heterozygosity and higher inbreeding were found at the extremes of species distribution limits. No effect of elevation on MLH (*R* ^2^ = −.02, *p* = .61) or on inbreeding estimated as Fhat (*R* ^2^ = −.01, *p* = .41) was found.

We did not find private alleles in *A. aurea* ssp. *promontorii*, and only one private allele was found in *A. aurea* var. *capensis*, whereas 2781 private alleles were found in *A. aurea* ssp. *aurea*. When samples were partitioned according to the mountain range, we found within each sampled mountain range a considerable number of unique alleles: 290 private alleles were found for samples from Sierra La Laguna, and 239 alleles were found for samples from Sierra La Giganta (Tables S4 and S6). No private alleles were found at the site level. These findings aligned with the detected population structure and geography patterns of the Baja California Peninsula.

### 3.4 Species distribution modeling

The final ensemble models had an AUC of 0.94 and a TSS of 0.99 on average. These scores indicate that our final model had high accuracy in predicting the *A. aurea* sensu Webb and Starr (2015) distribution. The importance of the selected bioclimatic variables varied between the algorithms (Table S7). For instance, the main variable explaining the distribution of *A. aurea* sensu Webb and Starr (2015) was BIO‐15 (Precipitation seasonality), with importance scores ranging from 0.18 (in the case of RF) to 0.24 (in the case of GBM). The second and third best variables varied between models; for the RF, it was BIO‐16 (Precipitation of wettest quarter) and BIO‐18 (Precipitation of warmest quarter), and for GBM, it was BIO‐10 (Mean temperature of warmest quarter) and BIO‐03 (Isothermality). Nevertheless, the three most influential variables contributed only 44% (GBM) and 35% (RF) to the explanatory power of the model, indicating that we may have missed some important predictors of *A. aurea* distribution.

**FIGURE 7:**
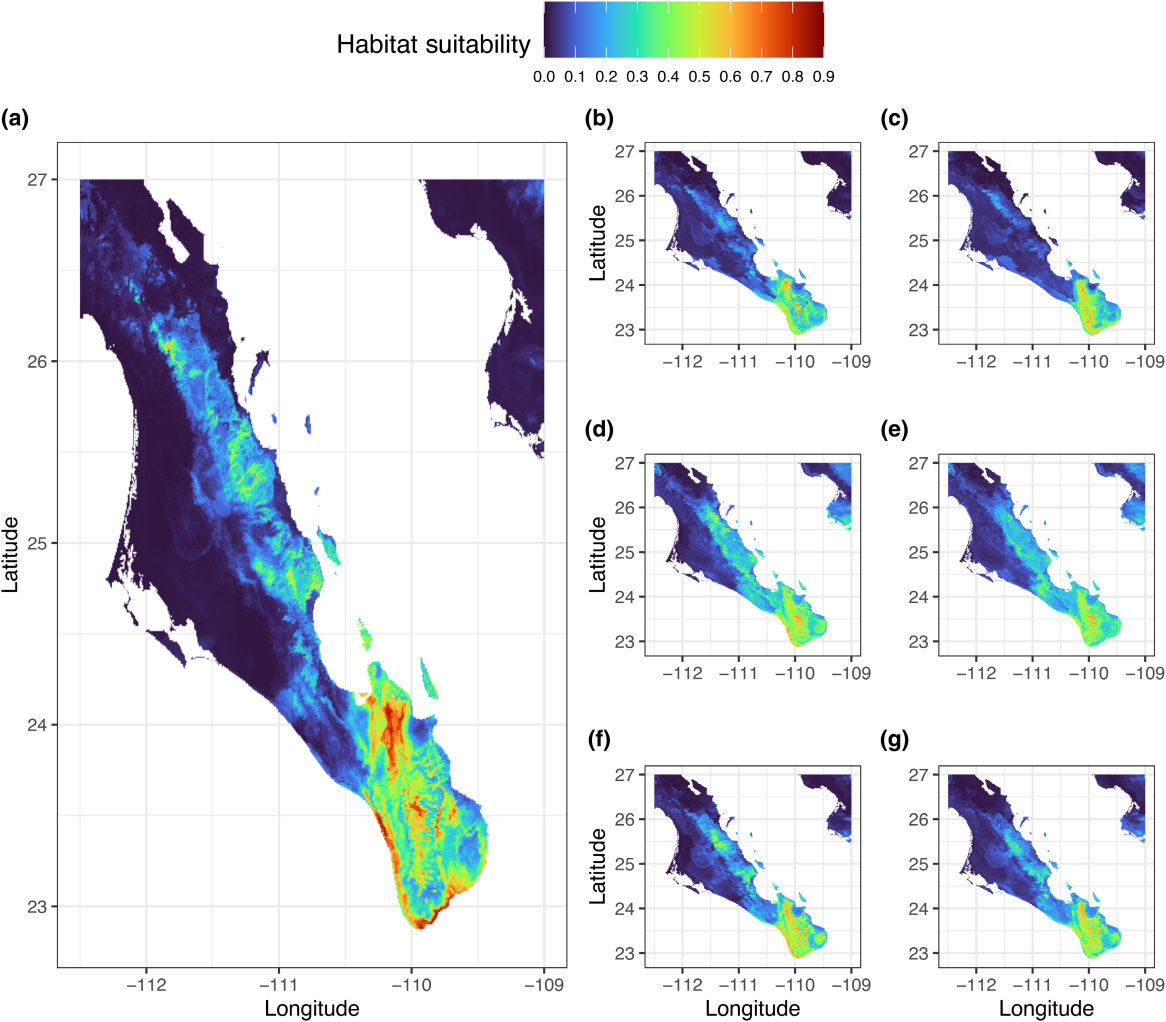
Current (a) and future (b–g) species distribution models (SDMs) and the respective spatial shifts for *Agave aurea* under different climate change scenarios and shared socioeconomic pathways (SSP 245 and SSP 585). (b, c) SDM for *A. aurea* under future climate scenario based on the ACCESS‐ESM1‐5 model under SSP 245 (b) and SSP 585 (c) in the years 2060–2080. (d, e) SDM for *A. aurea* under future climate scenario based on the MIROC6 model under SSP 245 (d) and SSP 585 (e) in the years 2060–2080. (f, g) SDM for *A. aurea* under future climate scenario based on the MPI‐ESM1‐2‐LR model under SSP 245 (f) and SSP 585 (g) in the years 2060–2080. Colors correspond to the high probability of species presence (orange and red) to the low probability (dark blue and blue).

The final model revealed that under the current climate, the areas having suitable and optimal conditions for the growth of *A. aurea* are the majority of the Cape Region, particularly the northern end of Sierra La Laguna, as well as the Pacific coast of the Cape Region (Figure 7a). These results are compatible with the real distribution of the species (Webb & Starr, 2015).

**FIGURE 8:**
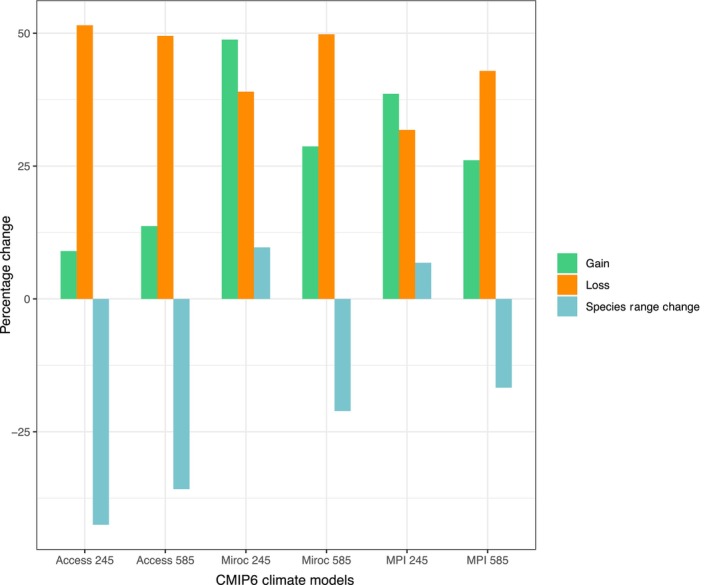
The percentage of distribution range change in *Agave aurea* sensu Webb and Starr (2015) under future climate change in 2060–2080 and different climate scenarios.

In general, the predictions of the ensemble models showed that there would be a decrease in the habitat suitability for *A. aurea* sensu Webb and Starr (2015) under future climatic scenarios. However, there were considerable differences between models and SSPs in the percentage of habitat change (Figures 7 and 8). The ACCESS‐ESM1‐5 model produced the most catastrophic results, with the highest habitat loss, whereas MIROC6 (SSP 245) and MPI‐ESM1‐2‐LR (SSP 245) projected a slight gain in available habitat for *A. aurea* sensu Webb and Starr (2015). The results indicated that by 2061–2080, *A. aurea* sensu Webb and Starr (2015) will undergo significant range changes from as high as −42.5% under SSP 245 (ACCESS‐ESM1‐5) to a slight gain in the range (+9.7%) under SSP 245 (MIROC6) (Figures 7 and 8). Except for the MPI‐ESM1‐2‐LR and MIROC6 under the medium pathway (SSP 245), all models agreed that there would be a reduction in suitable areas for *A. aurea* sensu Webb and Starr (2015) from −16.7 to −42.5% when compared to currently suitable habitat (Figures 7 and 8). The observed discrepancies among climate models are expected and result from different *initial conditions*, different *parameterizations* of interactions between Earth's land, ocean, cryosphere, atmosphere systems, anthropogenic activities, and different future emissions assumptions (Merrifield et al., 2023; Sanderson et al., 2015).

All models agreed that the areas likely to become unsuitable in the future include a significant part of Sierra La Giganta (that currently has genetically unique populations) and, to some extent, the Pacific coast of the Cape Region (Figures 7 and 8). In contrast, only a tiny portion of the currently unsuitable areas will become increasingly suitable compared to current habitat suitability. These expanding suitable areas would include a portion of the upper part of Sierra La Laguna, indicating that the species would probably migrate upward (Figure 7).

During the Mid‐Holocene (about 6 kya), the predicted geographical distribution of *A. aurea* was narrower than the contemporary distribution, with favorable habitats situated primarily in the Cape Region, especially along the Gulf of California coast (Figure S5). Further back in time, during the Last Glacial Maximum (about 22 kya), *A. aurea* appears to have experienced poor ecological conditions, as BIOMOD2 identified very low habitat suitability in the study area for this species (Figure S5).

## 4 DISCUSSION

### 4.1 Relationships within *A. aurea* sensu Webb and Starr (2015) complex

The first aim of our study was to analyze, using genomic markers, the relationships among *A. aurea* subspecies. We found mixed support for currently recognized taxonomic groups, with generally shallow genetic differentiation among morphologically recognized varieties. For *A. aurea* var. *capensis*, there was a disagreement among the analyses we conducted; some (i.e., TESS and PCA) indicated that only the samples collected at type locality Cerro de la Zeta (SLL_18) had unique genetic makeup and were differentiated from other sampling sites. In contrast, ADMIXTURE, fineradstructure, and the NJ tree grouped other southernmost sampling sites (e.g., SLL_3 and SLL_4) with *A. aurea* var. *capensis*, but each method grouped a different set of sites. Independent of clustering, the divergence of *A. aurea* var. *capensis* from other varieties was low, with only one private allele. The low number of private alleles may also be explained by the relatively low sample size for *A. aurea* var. *capensis*.

Furthermore, we found no evidence of divergence between *A. aurea* ssp. *promontorii* and *A. aurea* ssp. *aurea*: all methods clustered *A. aurea* ssp. *promontorii* with samples collected in and around Sierra La Laguna. Nevertheless, only one population of *A. aurea* ssp. *promontorii* was sampled, and it is not impossible that nonsampled individuals located at higher elevations may present a different genetic composition. However, samples collected at lower elevations around the mountain range of Sierra La Laguna were mainly found within or at the borders of arroyos (dry streams) and may represent plants whose seeds were dispersed by water pulses from higher elevations. Although the seed dispersion mechanisms in *A. aurea* are still unknown, water pulses are important for seed dispersion of other BCP plants (such as *Brahea armata*) and may have a strong effect (Wehncke et al., 2009).

Our data, therefore, lead us to conclude that *A. aurea* is more likely to represent several closely related genetic populations than separate species or varieties/subspecies. Our results agree with the study on the *Agave deserti* complex, where a low correlation between current taxonomy and genetic differentiation was found (Navarro‐Quezada et al., 2003). Moreover, our genetic data align with a previous morphological revision of *A. aurea* by Webb and Starr (2015), who found that these subspecies were very similar in vegetative characteristics, differing primarily in size and propensity to offset or remain solitary. Additionally, the hybridization between subspecies seems to be common, particularly in the southern part of the distribution range (Gentry, 1978). Nevertheless, further studies that would include more samples of *A. aurea* ssp. *promontorii* from higher elevations will be needed to decide on the taxonomic status within *A. aurea* conclusively.

### 4.2 Patterns of fine‐scale population genetic structure

*Agaves* are an intriguing arid‐adapted group of species that provide a unique opportunity to study the influence of multiple potential factors (i.e., geological and ecological) on plant population structure and diversification in the heterogeneous environment of the BCP (Eguiarte et al., 2021; Gentry, 1978; Webb & Starr, 2015). Nevertheless, only one previous genetic study was carried out on BCP's agaves, and it was mainly focused on unraveling phylogenetic relationships within *A. deserti* species complex (Navarro‐Quezada et al., 2003). Here, we generated over 10 K genome‐wide SNPs for *A. aurea* sensu Webb and Starr (2015), which allowed us to uncover, in some cases, unexpected patterns of fine‐scale differentiation.

We found evidence for three main genetic groups within *A. aurea* sensu Webb and Starr (2015), with previously unreported genetic separation between the two main mountain ranges in the region, i.e., Sierra La Laguna vs. Sierra La Giganta. Nevertheless, the genetic divergence among the identified groups was relatively low (*F*_*ST*_ = 0.03), consistent with the generally shallow population genetic structure found in other Agavoideae (Eguiarte et al., 2013). For example, *Yucca schidigera* populations in the BCP (*F*_*ST*_ = 0.067), as well as the endemic *Yucca capensis* (*F*_*ST*_ = 0.02) (De la Rosa‐Conroy et al., 2019; Luna‐Ortiz et al., 2021). Moreover, two subspecies of *Agave cerulata* from the north of the BCP also showed low genetic differentiation (*F*_*ST*_ = 0.098; Navarro‐Quezada et al., 2003), as did populations of *Agave palmeri* in Arizona (*F*_*ST*_ = 0.04; Lindsay et al., 2018), *Agave angustifolia*, in the Sonora state of Mexico (*F*_*ST*_ = 0.076; Klimova, Gutiérrez‐Rivera, et al., 2022), and *Agave potatorum* in southern Mexico (*F*_*ST*_ = 0.08; Ruiz‐Mondragón et al., 2023).

The shallow genetic differentiation found within *A. aurea* sensu Webb & Starr (1985) coincides with the *Agave* life history: outcrossing breeding system, long generation time, possibility of clonal reproduction, and involvement of long‐distance pollinators (bats and birds) (Eguiarte et al., 2013, 2021). Apparently, nothing is known yet about seed and pollen dispersal in *A. aurea* sensu Webb and Starr (2015). Nevertheless, within the range of *A. aurea*, the nectar‐feeding bat *Leptonycteris yerbabuenae* can be found (Arteaga et al., 2018). This bat species is regarded as the most important pollinator for the majority of *Agaves* (Fenster et al., 2004; Flores‐Abreu et al., 2019; Trejo‐Salazar et al., 2023), and it is a possible pollinator of *A. aurea*. Moreover, *L. yerbabuenae* on the BCP represents one panmictic population (Arteaga et al., 2018), suggesting that individuals can move over long distances, carrying pollen, homogenizing populations, and reducing the effects of genetic drift and selection in agaves. Nevertheless, studies on pollen and seed dispersal in agaves on BCP will be needed to understand better the drivers behind the observed population genetic structure.

Interestingly, several animal species display a genetic split roughly north of La Paz city (Dolby et al., 2015; Riddle et al., 2000), similar to the one found in *A. aurea* sensu Webb and Starr (2015). This genetic split has been explained by one of the major vicariance events on the BCP, the temporary isolation of southern Baja (Cape Region) from the rest of the Peninsula, owing to oceanic inundation of the Isthmus of La Paz ca. 3 Ma (Riddle et al., 2000). Nevertheless, the distribution patterns of many plant species do not agree with this hypothesis (Arteaga et al., 2020; Garrick et al., 2009; Gutiérrez‐Flores et al., 2016; Klimova et al., 2018), suggesting more recent ecological events related to Quaternary climate fluctuations (Araya‐Donoso et al., 2022). Due to the low divergence between *A. aurea* sensu Webb and Starr (2015) on each mountain range, we argue that the split is unlikely to have been caused by the million‐year‐old inundation of the Isthmus of La Paz.

The divergence in *A. aurea* sensu Webb and Starr (2015) may have resulted from more recent climatic conditions on and around each mountain range. Based on biotic characteristics, Sierra La Giganta and the Cape Region (i.e., Sierra La Laguna and surrounding areas) are considered two different ecoregions (De La Luz et al., 2008; González‐Abraham et al., 2010), each with characteristic flora and climatic conditions. Moreover, Sierra La Giganta is surrounded by extremely arid sandy low‐elevation desert areas of the Magdalena Plains, which may act as a barrier to the dispersal of genes and for the establishment of seedlings. Populations of *A*. *aurea* sensu Webb and Starr (2015) in Sierra La Giganta are scattered and can only be found at hilltops or near arroyos (Author's observation), which may further preclude connectivity among geographic regions. We also found a significant relationship between genetic distance and temperature (particularly temperature seasonality), which suggests divergence among samples on each mountain range and local adaptation. Further studies should delve into the genomic divergence among the *A. aurea* populations and search for the particular loci that may contribute to the observed differentiation pattern.

### 4.3 Genomic diversity and possible route of range expansion

Quaternary glacial–interglacial climate cycles with significant temperature and precipitation changes have resulted in species distribution shifts across the globe (Hewitt, 2000, 2004). This is especially true for plants, given that their distributions, phenologies, and physiological tolerances can be strongly tied to precipitation or the frequency and severity of winter frosts (McAuliffe & Van Devender, 1998; Van Devender, 2021).

In BCP, a dramatic change in floral composition happened since the Last Glacial Maximum (LGM; ca. 21 kya) (Butterfield et al., 2019; Dolby et al., 2015; Van Devender, 1977), when a cooler and wetter environment began to transition to warmer and drier conditions, and species once widespread in the lowlands followed favorable habitat, moved up in elevation and latitude, or sheltered in scattered oases (Butterfield et al., 2019; Grismer & McGuire, 1993; Klimova et al., 2017; McAuliffe & Van Devender, 1998). Novel arid‐adapted communities replaced mesic woodland vegetation (Van Devender, 1977). Mid‐Holocene range shifts of Sonoran Desert communities recognizable from plant macrofossils in packrat middens (Van Devender et al., 1994), and genetic data of desert plant species provide strong evidence of southward and northward expansion from refugia (Clark‐Tapia & Molina‐Freaner, 2003; De La Rosa‐Conroy et al., 2019; Garrick et al., 2009; Nason et al., 2002).

Range expansions are usually described by founder effects, where a few individuals (the founders) leave a source population, colonize a new neighboring area, expand, and send further founders. This repeated process leads to reduced genetic diversity along the expansion axis (Austerlitz et al., 1997; Slatkin & Excoffier, 2012). Our SNP data for *A. aurea* sensu Webb and Starr (2015) support the assumption that range expansion has played an important role in shaping spatial patterns of intraspecific diversity. However, the northward expansion along the BCP inferred for two columnar cacti (Clark‐Tapia & Molina‐Freaner, 2003; Nason et al., 2002) was not seen in *A. aurea* sensu Webb and Starr (2015), nor did we observe two expansion events from different refugia, as was inferred for the desert *Euphorbia lomelii* (Garrick et al., 2009). The decrease in diversity with increasing and decreasing latitude suggests both southward and northward expansions of *A. aurea* sensu Webb and Starr (2015) from a single refugium, located presumably in the northern part of the Cape Region, an area with high genetic diversity, low inbreeding, and suitable ecological conditions according to SDM. The best model explaining diversity distribution (MLH and inbreeding index) in *A. aurea* sensu Webb and Starr (2015) was a quadratic model with the highest diversity and the lowest inbreeding concentrated approximately between latitudes 24 and 25. From there, the diversity steadily decreased toward the north and south.

Further support for the above scenario comes from the species distribution modeling analysis. The observed reduction in genetic diversity is located within an area where available suitable habitat has increased since the LGM (22 ka) and Mid‐Holocene (~ 6 kya). Both lines of evidence, genetic and climatic, suggest a recent bidirectional range expansion of *A. aurea*.

### 4.4 Climatic future for *A. aurea* sensu Webb and Starr (2015) and conservation implications

Climate change is expected to shift plant distribution as species expand to newly favorable areas and decline in increasingly hostile locations. Ecosystems whose functioning is mainly driven by precipitation should be particularly vulnerable to climate change (Tompkins & Adger, 2004). Arid regions represent the best example of highly vulnerable ecosystems because warming may drive plant species to their physiological limits, and a decrease in precipitation will aggravate such effects. Indeed, global assessments have ranked deserts and semideserts at the forefront of vulnerability to global climate change (Sala et al., 2000; Mirzabaev et al., 2019).

Although authors like Tielbörger and Salguero‐Gómez (2013) argue that adaptations to lack of water and high temperatures commonly found in desert plants may result in the resilience of dryland species to climate change, the current evidence of the Sonoran Desert vegetation is telling a contrasting story (Hantson et al., 2021). A significant decline in the Normalized Difference Vegetation Index (NDVI), vegetation cover, community changes, and species distribution shifts have been observed, with the most striking changes being recorded in the lowland desert area (Hantson et al., 2021; Madsen‐Hepp et al., 2023). Moreover, other drivers of global change, such as overgrazing by free‐roaming livestock, mismanagement practices in agriculture, and man‐induced desertification, continuously increase the pressure on arid ecosystems and may lead to irreversible degradation (Carboni et al., 2023; Oswald & Harris, 2023; Reynolds et al., 2007; Thornton et al., 2009).

Under almost all climate change scenarios analyzed, the suitable habitat for *A. aurea* sensu Webb and Starr (2015) is expected to be reduced; this trend is particularly notable under the high‐end SSP 585. There were exceptions in SDM related to particular models (MPI‐ESM1‐2‐LR and MIROC6) and the relatively optimistic SSP 245, where *A. aurea* were slightly gaining new habitat (~8%). Nevertheless, we predicted that, on average, by 2070, *A. aurea* would lose over 20% of its currently available habitat. Our results are consistent with the proposed hypothesis that warming temperatures and increased water limitation negatively affect desert‐adapted species (Bombi et al., 2021; Hantson et al., 2021; Vale & Brito, 2015).

Moreover, climate change is also predicted to alter plant–plant and plant–pollinator interactions, which are essential for agaves (Blois et al., 2013; Creech et al., 2023; Gómez‐Ruiz & Lacher, 2019). Nurse plants are crucial for the establishment of *agave species* (Rangel‐Landa et al., 2015), with germination, growth, and survival positively affected by the presence of a nurse plant (Franco & Nobel, 1988; Rangel‐Landa et al., 2015). Meanwhile, the disruption of plant–pollinator interactions may have a negative effect on the sexual reproduction, genetic variability, and differentiation of *A. aurea* sensu Webb and Starr (2015), increasing its vulnerability (Gómez‐Ruiz & Lacher, 2019).

Currently, agaves experience a diverse range of threats. In many areas, the predominant danger is the direct human extraction of wild agaves used as raw material for alcoholic beverage (mezcal) production. Moreover, habitat degradation, land use change, and agriculture are considerable threats to agaves, affecting species in large parts of Mexico (Delgado‐Lemus et al., 2014; Tetreault et al., 2021; Valiente‐Banuet, 2023). On the BCP, due to historically low population density, agaves used to enjoy relatively low anthropogenic pressure. Nevertheless, our results showed that future climates of hotter and more arid conditions would not appear to favor *Agave aurea* sensu Webb and Starr (2015) as a considerable part of the species’ currently favorable habitat is projected to disappear.

Our study provides the first report on the population genomics and species distribution modeling information in *A. aurea* sensu Webb and Starr (2015), which may be used in conservation and management. We propose to consider the three identified genetic groups as separated genetic units or management units: the southernmost populations, the plants from Sierra La Giganta, and plants from the Cape Region distributed on and around Sierra La Laguna (Moritz, 2004). This information is particularly important for the southernmost and northernmost populations. First, these groups have lower genetic diversity and increased inbreeding. Moreover, the southernmost populations are under the heaviest anthropogenic impact, as they are located in an area of fast urban development. On the other hand, the northernmost populations are less abundant and, based on our data, are vulnerable to climate change. Considering how little is known about *A. aurea* sensu Webb and Starr (2015), conservation actions are urgently needed to protect this species. Additionally, more research is necessary to understand isolation barriers and factors governing this species' genomic structure and diversity. For example, an outlier analysis may point to the genomic regions involved in the observed pattern of geographic structuring and isolation‐by‐environment (IBE).

## AUTHOR CONTRIBUTIONS

**Anastasia Klimova:** Conceptualization (equal); data curation (equal); formal analysis (lead); investigation (equal); methodology (equal); software (equal); visualization (equal); writing – original draft (lead); writing – review and editing (equal). **Jesús Gutíerrez‐Rivera:** Data curation (equal); methodology (equal); resources (equal); writing – review and editing (equal). **Alfredo Ortega‐Rubio:** Funding acquisition (supporting); resources (equal); validation (equal); writing – review and editing (equal). **Luis E. Eguiarte:** Conceptualization (equal); funding acquisition (lead); project administration (equal); resources (equal); supervision (equal); visualization (equal); writing – review and editing (equal).

## FUNDING INFORMATION

This work was funded in part by project PAPIIT IG200122, UNAM, to Luis E. Eguiarte and Rafael Lira and by the operative budget of the Instituto de Ecología, UNAM.

## CONFLICT OF INTEREST STATEMENT

None declared.

## BENEFIT SHARING

Benefits from this research accrue from the sharing of our data and results on public databases as described above.

## Supporting information


Data S1:

## Data Availability

All of the genotypes are available from Dryad (DOI: 10.5061/dryad.0cfxpnw8t). Private access to download the data files URL: https://datadryad.org/stash/share/CzaRQZlC9yCxl_7MFJMo2WIlaOuZh9x2rrdPAPq8aAg.
