# Supplementary material for: Population genomics and distribution modeling revealed the history and suggested a possible future of the endemic Agave aurea (Asparagaceae) complex in the Baja California Peninsula
Source: Ecol Evol. 2024 Jul 24;14(7):e70027. doi: 10.1002/ece3.70027 (PMC11267983; doi:10.1002/ece3.70027)
Supplement: Supplementary file 1 — Data S1: [file ECE3-14-e70027-s001.docx]

Supplementary Information for

Population genomics and distribution modeling revealed the history and suggested a possible future of the endemic *Agave aurea* (Asparagaceae) complex in the Baja California Peninsula.

Klimova Anastasia, Jesús Gutíerrez-Rivera, Alfredo Ortega-Rubio, Luis E. Eguiarte

**Supplementary table S1.** Sites where 98 individuals of wild *A. aurea* from the Baja California Peninsula, Mexico, were collected, including information on geographical region, site name abbreviation, coordinates, and number of individuals used for genomic analyses (*N*). Samples were collected in 2023.

| Site | Lat | Long | Subspecies | Mountain Region | N |
| --- | --- | --- | --- | --- | --- |
| SLL_1 | 23.845796 | -110.276 | *A. aurea ssp. aurea* | Sierra La Laguna | 4 |
| SLL_10 | 24.078991 | -110.21127 | *A. aurea ssp. aurea* | Sierra La Laguna | 4 |
| SLL_11 | 24.107623 | -110.01772 | *A. aurea ssp. aurea* | Sierra La Laguna | 3 |
| SLL_12 | 23.095682 | -109.6205 | *A. aurea ssp. aurea* | Sierra La Laguna | 3 |
| SLL_13 | 23.984506 | -110.15264 | *A. aurea ssp. aurea* | Sierra La Laguna | 3 |
| SLL_14 | 23.526239 | -109.67783 | *A. aurea ssp. aurea* | Sierra La Laguna | 3 |
| SLL_15 | 23.538913 | -109.67541 | *A. aurea ssp. aurea* | Sierra La Laguna | 3 |
| SLL_16 | 23.812122 | -110.04872 | *A. aurea ssp. aurea* | Sierra La Laguna | 3 |
| SLL_17 | 24.130311 | -110.7578 | *A. aurea ssp. aurea* | Sierra La Giganta | 3 |
| SLL_18 | 22.881713 | -109.92848 | *A. aurea var. capensis* | Sierra La Laguna | 6 |
| SLL_19 | 23.528639 | -110.02656 | *A. aurea ssp. promontorii* | Sierra La Laguna | 5 |
| SLL_2 | 23.437928 | -110.23548 | *A. aurea ssp. aurea* | Sierra La Laguna | 3 |
| SLL_3 | 23.028175 | -110.02818 | *A. aurea ssp. aurea* | Sierra La Laguna | 3 |
| SLL_4 | 23.192402 | -110.108 | *A. aurea ssp. aurea* | Sierra La Laguna | 3 |
| SLL_5 | 23.428154 | -110.20153 | *A. aurea ssp. aurea* | Sierra La Laguna | 3 |
| SLL_6 | 23.630393 | -110.36065 | *A. aurea ssp. aurea* | Sierra La Laguna | 3 |
| SLL_7 | 24.024045 | -110.30216 | *A. aurea ssp. aurea* | Sierra La Laguna | 3 |
| SLL_8 | 24.12667 | -110.28977 | *A. aurea ssp. aurea* | Sierra La Laguna | 3 |
| SLL_9 | 23.855231 | -110.17626 | *A. aurea ssp. aurea* | Sierra La Laguna | 3 |
| SM_1 | 24.867588 | -111.06222 | *A. aurea ssp. aurea* | Sierra La Giganta | 3 |
| SM_10 | 25.942955 | -111.53281 | *A. aurea ssp. aurea* | Sierra La Giganta | 4 |
| SM_2 | 24.79485 | -111.09434 | *A. aurea ssp. aurea* | Sierra La Giganta | 3 |
| SM_3 | 25.869464 | -111.5483 | *A. aurea ssp. aurea* | Sierra La Giganta | 3 |
| SM_4 | 25.30403 | -111.20947 | *A. aurea ssp. aurea* | Sierra La Giganta | 3 |
| SM_5 | 25.36178 | -111.17364 | *A. aurea ssp. aurea* | Sierra La Giganta | 3 |
| SM_6 | 24.831623 | -110.80674 | *A. aurea ssp. aurea* | Sierra La Giganta | 4 |
| SM_7 | 24.832818 | -110.91514 | *A. aurea ssp. aurea* | Sierra La Giganta | 3 |
| SM_8 | 25.567523 | -111.28295 | *A. aurea ssp. aurea* | Sierra La Giganta | 4 |
| SM_9 | 26.054944 | -111.85339 | *A. aurea ssp. aurea* | Sierra La Giganta | 4 |

**Supplementary table S2**. Bioclimatic variables selected for modeling the distribution of *Agave aurea*.

| Bioclimatic variable | Units | Temporal scale |
| --- | --- | --- |
| Mean diurnal range (BIO 2) | Degree Celsius | Variation |
| Isothermality (BIO 3) | Dimensionless | Variation |
| Temperature Seasonality (BIO 4) | Degree Celsius | Variation |
| Mean Temperature of Warmest Quarter (BIO 10) | Degree Celsius | Quarter |
| Precipitation of Driest Month (BIO 14) | Millimeter | Month |
| Precipitation Seasonality (BIO 15) | Millimeter | Variation |
| Precipitation of Wettest Quarter (BIO 16) | Millimeter | Quarter |
| Precipitation of Driest Quarter (BIO 17) | Millimeter | Quarter |
| Precipitation of Warmest Quarter (BIO 18) | Millimeter | Quarter |
| Precipitation of Coldest Quarter (BIO 19) | Millimeter | Quarter |

**Supplementary table S3**. Mantel and partial Mantel tests summarizing relationships (*r* and associated *p* values) between genetic distance, geographic distance, and climate variables in *A.aurea, P-values*P<0.05;**P<0.01;***P<0.001*

|  | Mantel, r | Partial Mantel, r |
| --- | --- | --- |
| Geographic distance | 0.17* | NA |
| Elevation | -0.11 | -0.12 |
| Annual Mean Temperature (BIO1) | -0.05 | -0.05 |
| Mean diurnal range (BIO 2) | 0.25*** | 0.22* |
| Isothermality (BIO 3) | 0.16 | 0.16 |
| Temperature Seasonality (BIO 4) | 0.35*** | 0.33** |
| Max Temperature of Warmest Month (BIO5) | 0.2 | 0.18 |
| Min Temperature of Coldest Month (BIO6) | 0.25 ** | 0.21* |
| Temperature Annual Range (BIO7) | 0.34*** | 0.32** |
| Mean Temperature of Wettest Quarter (BIO8) | 0.89 | 0.11 |
| Mean Temperature of Driest Quarter (BIO9) | 0.13 | 0.13 |
| Mean Temperature of Warmest Quarter (BIO 10) | 0.06 | 0.08 |
| Mean Temperature of Coldest Quarter (BIO11) | 0.02 | -0.01 |
| Annual Precipitation (BIO12) | -0.03 | -0.02 |
| Precipitation of Wettest Month (BIO13) | -0.01 | -0.01 |
| Precipitation of Driest Month (BIO 14) | 0.07* | 0**.**03 |
| Precipitation Seasonality (BIO 15) | -0.13 | -0.16 |
| Precipitation of Wettest Quarter (BIO 16) | -0.03 | -0.03 |
| Precipitation of Driest Quarter (BIO 17) | 0.04 | 0.04 |
| Precipitation of Warmest Quarter (BIO 18) | -0.04 | -0.03 |
| Precipitation of Coldest Quarter (BIO 19) | 0.2 | 0.15 |

**Supplementary table S4**. Diversity estimates and corresponding standard deviation (SD) for three subspecies of wild individuals of *A. aurea* from the Baja California Peninsula genotyped with 10,765 SNPs.

| Subspecies | N | Fhat3 (SD) | MLH (SD) | F_is_ (SD) | Private alleles |
| --- | --- | --- | --- | --- | --- |
| *A. aurea var. capensis* | 6 | 0.18 (0.02) | 0.2 (0.01) | 0.19 (0.02) | 1 |
| *A. aurea ssp. promontorii* | 5 | 0.13 (0.05) | 0.21 (0.02) | 0.18 (0.06) | 0 |
| *A. aurea ssp. aurea* | 87 | 0.13 (0.05) | 0.22 (0.01) | 0.13 (0.05) | 2781 |

**Supplementary table S5**. Diversity estimates and corresponding standard deviation (SD) for 29 sampling sites of wild individuals of *A. aurea* from the Baja California Peninsula genotyped with 10,765 SNPs.

| Site | N | MLH (SD) | Fhat3 (SD) | F_is_ (SD) |
| --- | --- | --- | --- | --- |
| SLL_1 | 3 | 0.21 (0.002) | 0.12 (0.021) | 0.14 (0.008) |
| SLL_10 | 4 | 0.22 (0.005) | 0.09 (0.023) | 0.10 (0.021) |
| SLL_11 | 3 | 0.22 (0.006) | 0.09 (0.015) | 0.11 (0.023) |
| SLL_12 | 3 | 0.22 (0.007) | 0.12 (0.019) | 0.13 (0.026) |
| SLL_13 | 3 | 0.22 (0.013) | 0.09 (0.042) | 0.11 (0.053) |
| SLL_14 | 3 | 0.22 (0.003) | 0.13 (0.015) | 0.13 (0.013) |
| SLL_15 | 3 | 0.22 (0.003) | 0.11 (0.010) | 0.13 (0.014) |
| SLL_16 | 3 | 0.20 (0.023) | 0.20 (0.088) | 0.19 (0.091) |
| SLL_17 | 3 | 0.21 (0.004) | 0.15 (0.026) | 0.18 (0.016) |
| SLL_18 | 6 | 0.20 (0.006) | 0.18 (0.023) | 0.19 (0.013) |
| SLL_19 | 5 | 0.21 (0.016) | 0.13 (0.048) | 0.17 (0.064) |
| SLL_2 | 3 | 0.22 (0.004) | 0.10 (0.007) | 0.13 (0.017) |
| SLL_3 | 3 | 0.19 (0.006) | 0.22 (0.032) | 0.25 (0.023) |
| SLL_4 | 3 | 0.20 (0.017) | 0.16 (0.056) | 0.21 (0.068) |
| SLL_5 | 3 | 0.21 (0.004) | 0.12 (0.029) | 0.14 (0.016) |
| SLL_6 | 3 | 0.21 (0.005) | 0.11 (0.016) | 0.14 (0.021) |
| SLL_7 | 3 | 0.22 (0.005) | 0.10 (0.016) | 0.13 (0.019) |
| SLL_8 | 3 | 0.21 (0.007) | 0.11 (0.022) | 0.14 (0.028) |
| SLL_9 | 3 | 0.22 (0.016) | 0.12 (0.053) | 0.13 (0.065) |
| SM_1 | 4 | 0.22 (0.007) | 0.12 (0.011) | 0.11 (0.027) |
| SM_10 | 4 | 0.22 (0.002) | 0.17 (0.012) | 0.12 (0.007) |
| SM_2 | 3 | 0.23 (0.008) | 0.11 (0.044) | 0.09 (0.031) |
| SM_3 | 3 | 0.22 (0.007) | 0.15 (0.026) | 0.12 (0.029) |
| SM_4 | 3 | 0.22 (0.005) | 0.14 (0.017) | 0.11 (0.021) |
| SM_5 | 3 | 0.22 (0.004) | 0.14 (0.019) | 0.13 (0.015) |
| SM_6 | 4 | 0.22 (0.004) | 0.11 (0.015) | 0.10 (0.018) |
| SM_7 | 3 | 0.23 (0.001) | 0.11 (0.020) | 0.09 (0.003) |
| SM_8 | 4 | 0.21 (0.028) | 0.21 (0.124) | 0.14 (0.111) |
| SM_9 | 4 | 0.22 (0.004) | 0.15 (0.001) | 0.11 (0.018) |

**Supplementary table S6**. Diversity estimates and corresponding standard deviation (SD) for two main geographic regions (Sierra La Laguna and Sierra La Giganta) of wild individuals of *A. aurea* from the Baja California peninsula genotyped with 10.765 SNPs.

| Geographic region | N | Fhat3 | MLH | Fis | Private alleles |
| --- | --- | --- | --- | --- | --- |
| Sierra La Laguna | 63 | 0.13 (0.04) | 0.21 (0.01) | 0.15 (0.01) | 290 |
| Sierra La Giganta | 35 | 0.14 (0.05) | 0.22 (0.01) | 0.11 (0.04) | 239 |

**Supplementary table S7**. The relevance scores of the selected bioclimatic variables, both overall and by the algorithm.

| Model | Bioclimatic variables | Average Variable importance score among runs | SD |
| --- | --- | --- | --- |
| GBM | bio_15 | 0.00 | 0.00 |
| GBM | bio_10 | 0.01 | 0.01 |
| GBM | bio_3 | 0.02 | 0.01 |
| GBM | bio_4 | 0.05 | 0.02 |
| GBM | bio_2 | 0.05 | 0.02 |
| GBM | bio_16 | 0.07 | 0.02 |
| GBM | bio_19 | 0.07 | 0.02 |
| GBM | bio_18 | 0.10 | 0.03 |
| GBM | bio_17 | 0.10 | 0.03 |
| GBM | bio_14 | 0.24 | 0.04 |
| RF | bio_15 | 0.01 | 0.01 |
| RF | bio_16 | 0.03 | 0.01 |
| RF | bio_18 | 0.04 | 0.01 |
| RF | bio_3 | 0.05 | 0.01 |
| RF | bio_19 | 0.05 | 0.01 |
| RF | bio_2 | 0.07 | 0.02 |
| RF | bio_4 | 0.07 | 0.01 |
| RF | bio_10 | 0.08 | 0.01 |
| RF | bio_17 | 0.09 | 0.01 |
| RF | bio_14 | 0.18 | 0.02 |

**Supplementary figures.**

# Supplementary Figure 1.  A plot of ADMIXTURE cross-validation error and respective standard deviation based on ten repetitions for each K value, from *K*=1 through *K*=10, based on 98 *A. aurea* samples genotyped with 10,765 SNPs.

# Supplementary Figure 2. Pairwise *F*_ST_ differences among sampling sites of *A. aurea* from the Baja California Peninsula, Mexico*.* Colors represent *F*_ST_ values from the lowest of 0 in white to the highest of 0.257 in orange. Sampling sites are coded according to the mountain range and arranged from the southernmost to the northernmost; more information can be found in Supplementary table 1.

#

# Supplementary Figure 3.  A plot of TESS cross-validation error and respective standard deviation based on 30 repetitions for each K value, from *K*=1 through *K*=10, as based on 98 *A. aurea* samples genotyped with 10,765 SNPs.

# Supplementary Figure 4. Spatial genetic structure of *A. aurea* individuals from Baja California Peninsula, Mexico. Panels A and B. Clusters produced by the TESS analysis, in which each individual is assigned to the group with which it shares the most ancestrality (A, K=2 and B, K=3). Sampling sites were arranged from the southernmost (SLL_18) to the northernmost (SM_9). Panels C and D. Clustering results were plotted on a map colored according to the K=2 and K=3. The sampling site names coding corresponds to Fig.1 and Supplementary table 1.

**Supplementary Figure 5**. Current (A) and past (B-E) species distribution models (SDMs) and their spatial shifts for *Agave aurea*. (B and C), SDM for *A. aurea* under past climate scenario CCSM4 (B) and MPI-ESM-P (C) in the mid-Holocene (~6 K years ago). (D and E), SDM for *A. aurea* under paste climate scenario CCSM4 model (D) and MPI-ESM-P (E) in the Last Glaciation maximum (~22 K years ago). Colors correspond to the high probability of species presence (orange and red) to the low probability (dark blue and blue).
